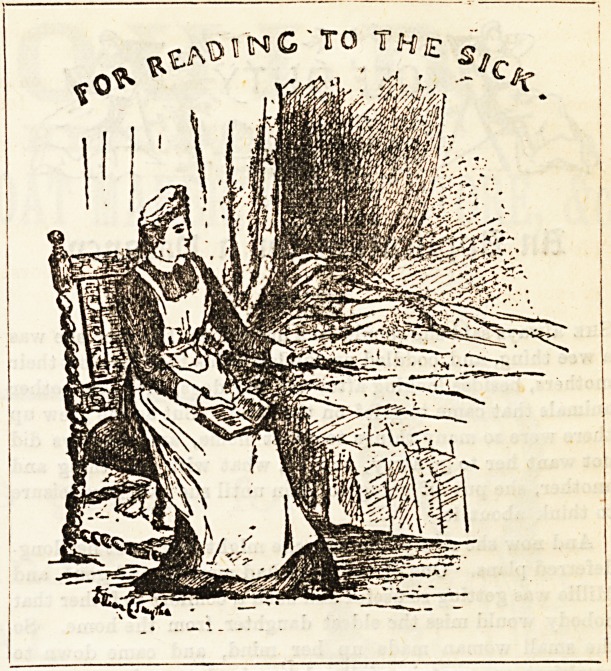# The Hospital Nursing Supplement

**Published:** 1892-08-13

**Authors:** 


### "The Hospital" Nursing Mirror.

Being the Extra Nursing Supplement of "The Hospital" Newspaper.

Contributions for this Supplement should be addressed to the Editor, The Hospital, 140, Strand, London, W.C., and should have the word "Nursing" plainly written in left-hand top corner of the envelope.

### En Passant.

A HOLIDAY IN SWITZERLAND.—"I think you will like to know that we have really started. We stayed a day in Antwerp, saw the pictures and Cathedral, and went on to Brussels, and thence to Berne, where we are now. The town is quite full, but we have rooms in a funny little hotel, and the weather is lovely. At Brussels, while we were in the Botanical Museum, we heard one little boy say to another, 'Here are some more English.' His friend answered, 'You don't know they are English.' 'Oh, yes, I do,' answered the boy, 'English always open their eyes so wide and their teeth are so clean!' We go on to Interlaken to-morrow."

SURREY CONVALESCENT HOME.—The anniversary meeting of this home, at Seaford, was very successful, and in his speech Sir Trevor Lawrence said that two hundred and twenty-seven Surrey men had derived great benefit from the home, and that one and all had expressed their gratitude for all the kindness and goodness shown them by the Matron, Miss Napper. Sir Trevor also appealed to the generosity of his audience to reward Miss Napper's success by complying with her request for an American organ, and so eloquently did he plead, that not only was the necessary sum for the organ forthcoming, but a substantial addition to the general fund as well. We congratulate Miss Napper on the success of her first year's work.

THE MARY ADELAIDE NURSES. — The twenty-fourth quarterly letter has been written to the nurses by Mr. F. R. Humphreys, at Hampstead. In it he draws attention to the immense field of study that lies open to infirmary nurses in the chronic cases with which they have so often to deal, and which are often looked upon as tedious and uninteresting, whereas when studied carefully they are an excellent education. There is no symptom in any disease without its reason, and it is only our ignorance or want of observation which prevents our understanding it. He urges all nurses never to lose an opportunity of studying and reading up the common diseases (the opposite of what is usually done), and points out that no nurse is worthy of her post if she does not do the best for each of her patients. "The image of God cannot be too carefully attended to."

THE SEA-SHELL MISSION.—We are asked to remind our readers of the existence of this society, and to suggest that contributions of shells are gladly received at 27, Benedict Road, Stockwell, London. Christmas cards are also begged, to be utilized for the children's scrap book mission. The latter aim is a very safe and pleasant outlet for patient energy, but the distribution of shells needs great discrimination on the part of the givers They are capital when supplied to divert weary children in out-patient departments of hospitals, or for convalescents or for poor bairns in workhouses, industrial schools, etc., but they should not be bestowed on really sick children or on any who are confined to bed, as a small fragment of one may easily cause the beginning of a bed-sore, which will be a grievous additional trial to the small sufferer. Shells and glass beads are amongst those offerings which are better omitted in wards for sick children.

VICTORIA HOSPITAL, HULL.—During the past month, those who wish to see women's labour and brain allowed to take the place it ought to have in the work and progress of the present day, have had more occasion than ever to rejoice. It is with much pleasure that we announce the appointment of Miss Annette Benson, M.B.B.S., London, a former student of the London School of Medicine for Women, and lately clinical assistant to the Paddington Infirmary, to the post of Resident Medical Officer to the Victoria Hospital for Children, Hull.

MASSAGE.—Among the numerous questions we receive, none are more numerous than those relating to massage. Our correspondents act as a very fair reflector of people's views on subjects various, but we could honestly wish that there were not such a prevailing longing to get "trained" either in general nursing, massage, or any other subject in "as short a time as possible." "How many lessons can I learn massage in," is a sample question, quite harmless in itself, but the desire to gain knowledge of a sort in as few hours as possible is not always as harmless. No woman who really means to be a thorough masseuse can become one without understanding elementary anatomy and physiology, and we have heard one of our best masseuses say that two years' experience is the least time in which a woman can be fitted for delicate work. Perfect training in massage takes three years under a medical man; the rubbing which satisfies many people is a very different matter, and can be learnt in as many months. It seems a pity that longer and more scientific training with daily practice cannot be managed somehow at many of the big hospitals; massage is, undoubtedly, at present often abused, it is very imperfectly understood by many people, and "trained masseuses" are being turned out with a rapidity which is, to say the very least of it, surprising.

SHORT ITEMS.—We much regret that by a mistake last week we spoke of "six" candidates who passed the L. O. S. examination from Manchester, whereas the number ought to have been seven.—Miss Pease, who has been Vice-Principal of Birmingham Training College for Elementary Teachers, is to go to Bristol to organise a similar institution.—The Brabazon Employment Scheme for paupers in workhouses is appreciated at Bradford; a sale of the work has resulted in a sum of £20.—Mrs. Catherine Loch, Superintenent Sister of the Indian Army Nursing Service, and Miss Ida Chinnery, Lady Superintendent of the Cama Hospital, Bombay, are both, says the *Queen*, contributing papers to the Nursing Section at Chicago.—Barnstaple is to have a district nurse at a salary of £80.—Coltishall Nursing Home has received a legacy of £50 from Mr. Thomas Carter.—The Winchester Hospital nursing staff have had a delightful day at Malshanger Park. They were all Mr. and Mrs. Wyndham Portal's guests.—Mary Florence Damsels, aged 25, late a hospital nurse (where trained is not stated) has been, says an Aberdeen paper, convicted of "serious fraud" in having gone to doctors and others and obtained money on false pretences; she has been sentenced to hard labour for three months.—Any donations for the St. Kilda Nurse Fund will be gratefully received by the St. Kilda minister, Rev. Angus Fiddes, and will be acknowledged in the *Glasgow Daily Mail*.—Miss R. A. Casson, assistant dispenser at the Birmingham and Midland Skin and Lock Hospital, has at the recent examination of the Pharmaceutical Society held in London, qualified successfully as a chemist and druggist.

### The Management of Consumptive Patients.

II.—DISINFECTION OF THE EXPECTORATION.

Bearing in mind the facts in the previous paper, it becomes incumbent upon the nurse to instruct her consumptive patient while he is yet under her care, so that on his discharge from the hospital he may carry away knowledge of the utmost importance, and faithfully follow out all he has been taught. The first and foremost care should be the expectoration. Each patient with cough and expectoration must be provided with a proper spittoon, and must be taught to rigidly adhere to the practice of spitting always and only into this spittoon. Now it has been shown that danger from the expectoration must be apprehended as soon as it is dry and converted into dust; so long as it remains moist little harm need be feared. This being so, it is necessary to always have some efficient disinfecting solution in the spittoon. The best of them all is a solution of carbolic acid of strength 1 in 20, or, if the smell of carbolic be objected to, a solution of perchloride of mercury of strength 1 in 500 may be used. Experience has shown that there is little risk of the patient drinking the carbolic by mistake in the night. The smell of the acid at once warns him of the danger. Besides the paramount importance of keeping the expectoration moist and disinfected by means of disinfecting solutions, there are other advantages arising from such a practice. If one goes into a ward where there are many consumptives, and where no disinfectant is used, a faint, sickly odour can at once be detected. This odour is not present where a carbolic solution is used. Again, it is so much more easy to clean the spittoons if the sputum is floating in fluid instead of closely adhering to the bottom and sides. The form of spittoon used is a subject worthy of consideration. It should be of the simplest description, so as to permit of easy and thorough cleaning. Earthenware is the best material. The vessel should take the form of an open-mouthed jar; there should be no rim or lid to it, or any ornamental processes to which the sputum would be likely to cling. The spittoon should be emptied, and the contents burnt at least twice a day. Unfortunately, it is too frequently the custom to throw the sputum down the sink or water-closet; in this case it is carried down the drains to the sewer or sewage farms and runs imminent risk at any time of its transit of becoming dry, whereas if it be burnt its power for evil is at once destroyed. The patient must be absolutely forbidden to spit, either on the floor or into his handkerchief and to this rule there should be no exception. Many patients after expectorating will take a handkerchief and wipe the remains of the expectoration from the lips; this should never be allowed, but in place of the handkerchief pieces of waste linen should be provided, and these after use burnt. All linen and bedding becoming soiled with expectoration should at once be cleansed in a strong solution of carbolic acid or perchloride of mercury. Special attention must be paid to the scouring of knives, forks, cups, and plates used by consumptive patients, and no one but the consumptive should in any case use his knives, forks, and cups unless one can be sure they are thoroughly cleansed and disinfected.

Since the tubercle bacilli are found in the dust of wards and rooms in which consumptive patients are confined, too much care cannot be bestowed on the dusting of the patients' room. The ordinary method of dusting a room will be calculated to do harm rather than good in this case, for to sweep the dust up into a corner with a dry brush, collect it in a dust-pan, and then throw it away, is simply to scatter to the winds a probable source of infection. A more approved way is to thoroughly sprinkle the floor with water, and then to search out all places in the room where dust is likely to accumulate, and remove it on a damp cloth or piece of waste linen; the linen and dust adhering to it may then be burnt. Important though all this may be, if the rule of spitting only into the spittoon, and nowhere else, be firmly enforced and carried out, these subsidiary regulations about the dust and soiled linen become less urgent, but to make all secure the nurse should look to the performance of all these duties.

Consumptives, in addition to having tubercular disease of the lungs, very commonly are also affected with tubercular ulcers in the intestines, so that another source of danger is from the excreta. These should be received into carbolic, and thoroughly disinfected before being thrown away. In connection with this subject it may be mentioned that patients are sometimes in the habit of swallowing the sputum instead of expectorating it; many in this way infect their intestines, so that apart from mere principles of cleanliness, on the grounds of danger alone, the patient should be advised not to continue the practice. By the observance of all these rules the nurse is gradually educating her patient up to a higher standard, and besides improving his own condition, reducing the danger he is to others to a minimum. Consumptives should be made clearly to comprehend the reason for all these directions; they should be given to understand that unless this is done they are liable to give their disease to their friends and all with whom they come in contact.

Next in importance to the disposal and disinfection of the expectoration, comes attention to the patient's surroundings, and more especially, to ventilation and warming. We are dealing with a disease which may lay fair claim to be called an infective disease. Now, the Local Government Board requires that infectious hospitals shall have enough space to allow in each ward 2,000 cubic feet per patient, and ventilation enough to permit of a supply of 3,000 cubic feet of air per hour to each patient. This applies to hospitals for scarlet fever, typhus, typhoid, diphtheria, and small-pox. But this regulation is just as necessary for wards and rooms in which consumptives are confined, as for those in which fever patients are treated. The consumptive patient has every need of an abundant supply of fresh air. In proportion to the extent of the disease of his lungs, so is his demand for an increased quantity of pure air. The larger the volume of air supplied to him, the less likelihood is there of danger to those whose duty it is to be with him, and the more diluted will be the poison emanating from him.

Again and again evidence has been forthcoming to show in a manner that does not admit of dispute, that the supply of fresh air is a mighty factor in diminishing the liability to consumption. In this matter of a due supply of fresh air, the nurse must be prepared to fight a battle with the consumptives, for they commonly suppose that because the chest is affected, it must be especially bad for them to come in contact with fresh air, on account of their supposed greater susceptibility to chills. It can frequently be extracted from a patient that when at home he rigidly confines himself to one room, takes great care to have all windows closed, and makes it his business to stuff up every crevice and crack through which the air could come. A large fire is also kept burning night and day. What wonder he has attacks of hæmoptysis, incessant cough, and becomes progressively weaker. To all complaints of chill and cold the nurse must turn a deaf ear. The very worst one can do for a consumptive is to keep him in a small room with little or no ventilation, and to see that the temperature keeps at from 70 deg. to 75 deg. It should be a rule, never to be departed from, that the temperature of the room or ward should never be allowed above 62 deg., and never below 58 deg., anything above 62 deg. being too hot, anything below 58 deg. too cold. The dangers and inconvenience arising from an overheated ward are manifest. In wards where the temperature is from 62 deg. to 58 deg. the patients are comfortable, sleep well, are rarely troubled with cough, and attacks of hæmoptysis are unusual; in a ward, on the other hand, where the temperature is 70 deg., these conditions are reversed, the cough becomes harassing, and hæmoptysis of frequent occurrence. With a view to ventilation, the door should never be closed, and twice during some part of the day the windows should be thrown wide open for an hour, and one window at least in every ward should be kept continually open night and day. For this purpose of continual ventilation opening the window an inch or two is quite sufficient.

### Our Indian Letter.

July 11th, 1892.

You all have been having a discussion in England as to how more private nursing would succeed in India, so now I send you some of my own observations on the subject.

That good nursing is needed in India there is no doubt, but that the services of good nurses would be sought for and properly paid there is very great doubt. In Bombay, Madras, and Calcutta there are already a number of well-trained nurses, and I doubt whether large establishments in any other towns would pay.

Every private nurse coming out to India should qualify as a midwife; monthly nursing and midwifery being the one branch that would succeed best, but a lady setting up in that capacity would have much less chance of being employed than a common woman, and there would be a great prejudice against an unmarried woman unless she was very elderly. Many ladies to whom I have spoken on the subject of having more private nurses sent out here, all assure me that nothing on earth would induce them to employ a gentlewoman, and men are much of the same mind. They all would prefer some motherly body, who would not expect to be more than a cipher in the domestic establishment; for there exists in India a very strong prejudice against "lady nurses" as private nurses.

A number of respectable wives of the soldiers train as nurses and midwives at the Eden Hospital, Calcutta; the General Hospital, Madras; and, I expect also, in Bombay. They are much preferred to other nurses; they are recommended by the ladies of the various regiments, by the staff surgeons, and are sure of some employment during the year from one month to, perhaps, six; it depends a good deal on what station they are in, and the number of married ladies in the station. They command, when employed, from 100 to 150 rupees a month; they generally fix their charges according to the rank of the husband whose wife they nurse.

Distances are very great in India, and there are some of these women in every station. Railway travelling is also very expensive, and people either nurse their own sick, make use of these regimental nurses, or employ the Eurasian nurses, and sometimes even the native nurses, who are trained in the Dufferin and general hospitals, where they exist. Nursing in the army to a great extent is provided for by the Indian Nursing Service and Lady Roberts' Auxiliary Staff. Officers can come into hospital, be nursed, doctored, and fed in their own wards for two rupees a day, and in emergency for nothing; and in their own quarters by the nursing sisters; so they are not likely to employ civil nurses. In most of the large towns there are paying wards in the civil hospitals, and many civilians go in there in preference to being nursed in their own home.

There are times when a nurse is most urgently wanted in some of the smaller places, but it would not pay a nurse to live there on the chance of that occasion arising. People here do not usually send for a nurse unless the patient is almost *in extremis*; in that case they would send to the nearest hospital, rather than run the risk of waiting a day or perhaps two days before the trained European nurse could arrive. If a nurse started a house, however small, she would scarcely get it under 25 rupees a month, she would have to have a servant always there to keep it, and unless she could cook her own food, she must have a cook, which would at least mean eight rupees more a month, and when living at home she would have to employ two other servants, that would be 10 rupees more.

The other alternative would be to live at an hotel, that would cost from five rupees a day. At one Hill Station I know it would cost eight rupees a day; in addition, there would be servants' fees and washing, and in the hot season, at least six annas a day for punkah coolies and four annas a day for ice; I know the little unavoidable extras added a rupee a day to my bills.

If she could meet with nice people who would board her when out of employment for two rupees a day she might get on, but she would require good social and financial recommendations for that. To ensure success she would have to know people who could recommend her, besides a number of surgeons, and the latter she would have to keep reminding of her existence, then she must advertise in the papers and distribute circulars, and all this would cost money.

I visited the lady doctor in charge of one of the Dufferin Hospitals the other day. From these hospitals they supply nurses for private work; they train Eurasians and native Christians, who have to pass examinations at the end of their course, and, according to merit, they receive first class and second class certificates; they are constantly asked for and employed. I suggested that it would not be a bad thing for the Dufferin Fund, if they boarded at their hospitals, for a fixed sum, European nurses who were private nursing for themselves. I said what a good thing it would be for the nurses, for they would see what was going on in the hospitals, and be brought in contact with medical men, who would afterwards recommend them patients. She did not like the idea at all, and she thought it would interfere with their nurses' interests. There is a number of convents and Sisterhoods throughout India and some of these supply nurses. I have been in no town yet without a convent. If a nurse cares to sink her social status and sink to the shopkeeper class and live with Eurasians, she might live for less, but her work would lie among them mostly; they would pay more certainly than the higher classes. Then, if she cared to nurse Parsees (natives of sorts), she might make a good deal of money, but she must beware of eating their food, it is cooked in such a disgusting fashion, I am told, by one who has nursed among them. I have heard of a widow lady, trained, certificated, and diplomaed, young and handsome, who, for the sake of her children, has sunk caste and lives and nurses among these classes. Her father was an officer; her former friends no longer know her, and her only companions are all of the shopkeeping and Eurasian classes, amongst whom her work lies principally.

What want there has been for professional nurses has now, more or less, apparently, been supplied. In the Hill Stations there are already one or two established, and they gravitate to the plains in the winter. One trained midwife and nurse I know, the wife of an army pensioner, has, on occasion, earned 200 rupees a month, but being out of employment some time she has been glad temporarily to accept the Matronship of a Women's Hospital on 20 rupees a month, with free quarters (not board), fuel, and light. Twenty rupees just now is equivalent to £1 5s. 10d. She has in her harvest times saved money, so, with her husband's pension, she makes both ends meet.

A lady, well connected and well trained, with both London and provincial hospital experiences, set up in one of our large towns as a private nurse; she had good introductions, but she soon had to give it up.

I met a lady nurse last year at one of the Hill Stations who has been nursing some time in India. I asked her opinion about private nursing in India. Her charges were five rupees a day and eight rupees where there was more than one patient, board, washing, and lodging, travelling expenses (first class), and wine. She was then out of employment. She told me that she did not consider nursing a paying matter if you were dependent on it; and that no woman could make it pay in India unless she had a home independent of her labours, which this nurse had. She said her earnings clothed her and furnished pocket money for her; but she was sometimes months out of employment, and at her last case they had not been able to pay her the full fees.

One nurse, a soldier's wife, looked after a lady in the Punjaub, who was much injured by an attack on her by a native; it was a trying case requiring much skill, as soon as the lady was well her husband took her home, promising to send the nurse her fees from England. That was some time ago, and she has not yet been paid.

So there is another difficulty to be faced. Many people are in debt in India; they blame it all to the depreciation of the rupee, but I think personal carelessness and extravagance goes a little towards it, and a nurse, with hasty journeys before her, requires ready money.

A nurse before venturing out here has a good deal to consider; if she does not understand Hindustani, she will certainly be robbed. She has to face the possibilities of ill-health or total breakdown, and then the awful expense of return passage home.

For nurses with money, who would come out and give their services, there would be plenty of work to be found here as anywhere else, but it is no place for fortune hunting.

There are a few nursing prizes in India; the Matron Superintendentship of the Madras General Hospital has just been filled up, the salary 300 rupees a month, free and furnished quarters, and a staff of 16 nurses. The appointment was in the hands of the Secretary of State, or, more literally, subject to his approval. I should say that that is the "first prize" in India, for it is a splendid hospital.

I hope you don't think I take a dismal view of things, but I state exactly how things have appeared to me, and send you my observations on the subject. I take an interest in this subject, for I had meditated starting a private nursing institution myself, and I see now what a folly it would be.

Two years ago a gentleman suggested my starting a private nursing establishment in Cairo; he considered there was an opening in the season; he knew nurses who commanded £5 a week there, and he said they could nearly always get a passage home with a patient at the close of the season. Perhaps some one who knows Cairo better than I do can give you some information about this idea, for I don't feel I know enough to write about it; but India I do know about. I have been a good deal through the country, and know, with the exception of the Madras Presidency, the nursing wants out here. O. N.

### Appointments.

Miss M. N. Didier d'Amblon, whose appointment as a County Council lecturer we announced last week, asks us to state that she was first probationer and then staff-nurse at Birmingham General Hospital, not charge-nurse as we stated. We regret that we mis-read her testimonials which she kindly sent us.

Sir Titus Salt's Hospital, Shipley.—Miss Kate Hay, of the Royal Infirmary, Windsor, has been appointed Matron-nurse of this hospital, in succession to Mrs. Wiseman, who has resigned. Miss Hay trained at Boston Hospital, Lincolnshire, and after gaining her certificate, she went to St. Mary's, Paddington, and from there to the National Hospital, Bloomsbury, where she received a certificate for massage and electricity. Miss Hay was head nurse at Windsor, and in charge of the male wards, and she was elected Matron at Shipley from a large number of candidates.

### Hospital Porters.

This general designation covers rather an extensive class, if we take it as including the men of great intelligence and a fair education combined with much technical knowledge, who wait on the surgeons in the operating theatre, as well as the lads who begin their career with the most humble and mechanical of tasks. The former have it in their power to become most valuable hospital servants, and generally develop a great enthusiasm for the instruments and appliances entrusted to their care. In large hospitals they have opportunity for becoming familiar with the very newest inventions in these lines, and are quite competent to judge of the probable success which will attend each novelty, which they criticise from their own practical standpoint. They know all the doctors' ways, and humour their fads, but variously. For instance, the great man whose marvellous successes have won him world-wide fame, is certain of getting not only service, but devotion, from his satellites; whilst the second-rate gentleman is treated to a far more limited deference. Perhaps the man with the shortest temper gets the promptest attention (as he apparently does all through life!), but devotion in the operating theatre is always commanded by successful skill.

Where there is a medical school, the students generally take a regular course of instruction in the use and names of the instruments from the guardian of those treasures, as a supplement to their usual lectures and before going up for certain examinations.

The porter at the hospital entrance is the one most familiar to the general public, and we are glad to note that he is often a courteous and agreeable personage, and it does him credit when he thus ensures to each visitor a pleasant impression of the tone of the institution. A surly gate porter should certainly be unknown; whatever other virtues he may possess they cannot cancel roughness or incivility to strangers who make shy or awkward enquiries, or, worse still, to the sick and suffering, whose poverty prevents their resenting insults.

At King's, St. Thomas's, the London, and the Royal Free we hear pleasant reports of the civility of porters to patients, but there are two or three small hospitals where applicants make grievous assertions regarding the rudeness of certain jacks-in-office.

In some out-patient departments an iniquitous system of "feeing" prevails, and those patients whom poverty or principle prevent from submitting to this imposition know perfectly well that their claims to see the doctors will be ignored by the man whom they have not tipped until all the others have been attended to.

A funny story was told by a woman who had to go every week for a long time to a special department at a hospital. She said she soon noticed that the porter had a friendly feeling for women with young children, and let them have the earliest interviews, "and so," quoth she, "as I didn't happen to have a baby of my own, I just borrowed one from a neighbour, and I managed all right after that."

The porters who receive and carry in the bad accident cases are often very skilful, and lift heavy and helpless patients with very little exertion or apparent difficulty. There are other duties of a more painful nature than any pertaining to the living, and those are offices connected with the dead. Unhappily, this kind of work has usually a hardening effect on the ignorant mind, and men get not only callous to many most unpleasant details, but they grow indifferent to the effect of such things on unaccustomed eyes.

They do not sufficiently consider the feelings of the friends of the deceased persons, and often take a kind of morbid satisfaction in the ghastly details, which might often be softened, if not altogether mercifully ignored. A very strict supervision of the mortuary arrangements, as well as of the behaviour of the people coming to remove the dead, is absolutely necessary, and it should be done by someone whose own position and demeanour are beyond suspicion. Also, this sad but needful department should make frequent changes in its officials, not feeling itself justified in allowing one porter to continue for any long period in attendance on work of so peculiarly trying a description. It is a temptation to retain a man in a position which he fills competently, but it is not always humane, and it would answer in the long run to economise the health of the individual, both of mind and body, by giving him at regular intervals a spell of less distressing employment.

Porters, like ward-maids, are supplied with distinctive uniform, but it varies according to their standing, and also according to the nature of their duties.

The porters who carry the provisions and stores to the wards are usually popular with the patients, and also, on their part, take a considerable amount of interest in the latter. Any accident or misfortune which has found its way into the papers seems especially to endear the victim thereof to a certain type of hospital official.

The man who brings in the coal in the early morning, and the man who distributes the letters and newspapers, soon become well-known to each patient, and it is pleasant to see the amount of trouble cheerfully taken by the latter to deliver the illegibly-addressed missive to the invalid to whom it is a welcome event to get news from his "own folks."

### Final Examinations of Nurses at the Johns Hopkins Hospital.

The final examinations of the second class graduating from the Training School for Nurses of the Johns Hopkins Hospital began in the first week of May and lasted throughout the month. The examinations were entirely written. Ten subjects were chosen by the Superintendent of Nurses from the schedule of lectures. The different specialists set the questions, five on each subject, and afterwards read and marked the papers. From two to three subjects were taken in a week, and from an hour to an hour and a half allowed to each examination. The class standing was 88·9. The subjects and questions were as follows:—

### First.—General Medicine.

1. What are the advantages and disadvantages of the different ways of taking the body temperature?

2. Typhoid Fever. Enumerate the symptoms at the beginning of the third week of a severe case. Describe your method of caring for the mouth and skin.

3. Define the following terms: Anæmia, anasarca, cyanosis, diuresis, diaphoresis, œdema, photophobia, hæmoptysis.

4. What are the general points to be noted when examining the pulse? How would you determine an "increase of tension"? What is a "dicrotic pulse"?

5. State briefly how you would take care of a case of complete paraplegia prior to the development of bed-sores.

### Second.—Materia Medica.

1. State briefly the treatment of a case of opium poisoning, and give the physiological antidote of opium.

2. Give the dose of strychnia, atropia, and morphia, and give the amount of morphia in Magenrie's solution and in the so-called U.S.P. solution.

3. How would you prepare a nutrient enema? Give contents, amount, mode of administering it.

4. Mention three emetics among which must be one that is given hypodermically.

5. Give two preparations of digitalis, with their average doses, and state very briefly what you know about the use and the action of digitalis; and what symptoms you have to report to the physician after prolonged use, as probably indicating a toxic effect.

### Third.—Surgery.

1. Describe in detail the manner in which you would prepare a patient for amputation of the thigh.
2. A patient has received a severe lacerated wound of the hand; state how you would treat the wound while waiting for the surgeon, and your reasons for so doing.
3. A patient has sustained a simple fracture of the leg, a surgeon cannot be obtained for several hours. What would you do to render his condition more comfortable in the meantime?
4. State the characteristics of arterial, venous, and capillary hæmorrhages. Give the distinguishing characteristics of malignant and benign growths.
5. Describe the changes occurring in a wound healing by granulation.

### Fourth.—Urinalysis.

1. What is the normal quantity of urine passed in 24 hours? Would you expect to find any sediment in normal urine, if so, how much?
2. Under what circumstances would you expect to find an increased quantity and a diminished quantity?
3. You have charge of a pneumonia case in winter-time, and notice that the urine is scanty and of a high colour, and that it has a thick salmon-coloured sediment, what inference would you draw? Is the condition alarming?
4. Give two tests for albumen; for sugar. What precaution would you take in albuminous urine before testing for sugar?
5. What conditions of the urine would lead you to suspect carbolic acid poisoning?

### Fifth.—Hygiene.

1. Describe how a sick-room should be cared for.
2. Disinfection of a typhoid stool. Give the best method and the objections to the use of certain agents which are at times employed.
3. How and why should tuberculous sputum be disinfected?
4. Give a few simple ways of securing proper ventilation in the sick-room.
5. Supposing you have been nursing a scarlet fever patient, what precautions would you take as regards your person before attending another patient?

### Sixth.—Obstetrics.

1. What are the signs of pregnancy? What signs at an early date are presumptive of pregnancy, and what later signs are infallible evidences of the existence of pregnancy?
2. How calculate the stage of a given case of pregnancy, and how reckon the probable date of confinement?
3. What immediate preparations is it necessary to make for confinement?
4. How will you care for the mother in the puerperal weeks after the child is born?
5. What are the signs of danger after the confinement, and how may you be the means of introducing some of these troubles?

### Seventh.—Gynæcology.

1. Enumerate the pelvic bones and their relations. What are the functions of the ligaments of the pelvic bones? Of what does the uterus consist? Describe its relation to the bladder, rectum, and vagina. What is the broad ligament, and what structures does it contain?
2. Describe briefly the preparation of a patient for a minor gynæcological operation. What precautions are to be taken in using the catheter, also in giving an enema after a perineal operation? If a vaginal pack is used, when should it be removed?
3. What are the different douches employed, and how administered? If hæmorrhage follows a cervical of perineal operation, what treatment should be carried out?
4. Define the terms pyosalpinx, hydrosalpinx, salpingitis, and ovaritis.
5. How would you prepare a patient for coeliotomy (abdominal section)? and give an outline of the after-treatment.

### Eighth.—Children.

1. State the significance of the fontanelles or soft spots in the infant's head, their time of their closure, and the influences which may affect the time of closure.
2. The object of the umbilical cord. How should it be dressed?
3. Which secretions of the digestive apparatus are deficient in the new-born, and what indication has this deficiency upon the diet?

4. Give the intervals for nursing infants at one month and six months of age. When is the best time of day for bathing infants? Temperature of the bath?

5. How much sleep does the new-born infant require? Give the main principles in the clothing of infants.

Ninth.—Dietetics.

1. Select any one of the following soups: celery, mock bisque, or potato. Give the recipe in detail.

2 (a) Select from the list of puddings given in the course in cooking any one that you please. Give a recipe for it. (b) State to what extent it is nutritious, and why?

3. (a) What is the chief value of tea and coffee to the world? (b) Under what circumstances may they be injurious?

4. (a) Into how many and what classes is food divided? (b) What are carbohydrates? (c) Give the composition of cow's milk. (d) What is the percentage of starch in bread? (e) What is the percentage of fat in the cocoa bean?

5. In what way does food supply the wants of the body?

Tenth.—Massage.

1. How would you apply massage as an aid to digestion?
2. What are the contra-indications to the use of massage?
3. Enumerate different methods of applying massage.
4. What distinction would you make in giving massage in the morning and in the evening? and why?
5. Describe in detail method of applying massage to the back.

The Commencement Exercises.

The graduating exercises of the school took place on June 3rd, at four o'clock, in the rotunda of the hospital, which was, as before, beautifully decorated with plants and flowers.

*Order of Exercises.*

Music—March from "Lenre."

Prayer—The Rev. John B. Harding.

Annual Report of the Training School—Miss Isabel A. Hampton, Principal of the Training School.

Address to Graduates—Dr. Howard A. Kelly.

Address—D. C. Gilman, President of Johns Hopkins University.

Presentation of Diplomas—Dr. Henry M. Hurd, Superintendent of Johns Hopkins Hospital.

Names of Graduates.—Helena Barnard, Minnie James, Ada H. Patterson, Anna C. Jack, Clara E. Worthington, Lucy P. Welch, Estelle Hall, Evvy E. Smith, Katharine M. Laing, Marion G. Hemming, Lucy Ashby Sharp, Mary L. Chamberlain, Emily J. Macdonnell, Annie C. MacRae, Fanny Priestly Toulmin, Louise K. Rudolph, Mary H. Townsend, Helen B. Higbee, Lilian J. T. Sills, Margaret R. Richardson, Sarah L. Tarleton.

### Presentation.

Cumberland Infirmary, Carlisle.—On August 5th, Miss C. A. Allen was presented with a silver afternoon tea service, in a handsome leather case, on the top of which was a silver plate bearing the following inscription:—"Presented to 'Sister Ruth,' Matron of the Cumberland Infirmary, Carlisle, by the Nursing Staff, as a token of their love and gratitude." This was also accompanied by a letter signed by all the nurses, expressing their thanks to Miss Allen for all she had done to make them happy and comfortable. At the same time, Miss Allen was also presented with a handsome carved oak tray, with silver handles, in the centre of which was a silver plate bearing the following inscription:—"Presented to Miss C. A. Allen by the present and past House Surgeons, on the occasion of her leaving the Cumberland Infirmary." Miss Allen goes to Birmingham early in September.

### Wants and Workers.

The Matron of the Ledbury Cottage Hospital will be very grateful if some reader of The Hospital will kindly give her an old piano for the above institution, to cheer the patients in their hours of pain and weariness.

### Everybody's Opinion.

[*Correspondence on all subjects is invited, but we cannot in any way be responsible for the opinions expressed by our correspondents. No communications can be entertained if the name and address of the correspondent is not given, or unless one side of the paper only be written on.*]

### "THE MEDICINE AS BEFORE."

"Sister Ethel" writes: May I be permitted, through the medium of your columns, to protest against the very unsafe practice of labelling medicine or lotion bottles, "The mixture as before," or "The lotion as before," as the case may be. To look at it from the patient's point of view. It is unsafe, because if the sufferer is unable to afford the luxury of a trained nurse, he or she is at the mercy of one of the family, or, may be, a friend who comes to help with the nursing. Seeing bottles labelled, "The mixture as before," and having very vague ideas as to what the previous directions were, the medicine is given at any time that it happens to be remembered, in utter disregard of the strength of the mixture, the effect of it upon food, or the proper time for it to be taken. Or, supposing a patient is supplied with a trained nurse, who must, of course, take a certain number of hours daily for sleep and exercise. Unless she is very particular to leave clear directions to her substitute about the hours for medicines, lotions, &c., they are either given or used at will, or disregarded altogether because the substitute is in complete ignorance of what the "as before" alludes to. Or it may so happen that more than one lotion is required, and in that case, if both bottles are labelled "as before," it is very puzzling. Is it fair for a nurse who takes up a case from another nurse to be left to puzzle over what "as before" refers to; or can she be expected to remember after one visit from the doctor all his directions as to the various times for lotions and medicines, &c., and it is quite probable that he will end his remarks with, "You will find all the bottles marked," and she often finds nothing more definite on the label than, "The mixture as before."

### MALE NURSES.

"E. B." writes: In your article on the Hamilton Association that appeared in your paper of July 2nd, you complain of the higher fee of the Hamilton Association as being exorbitant. I, like many others, fail to see this. A valet cannot be classed on the same level as a good trained nurse, and a nurse's work is not constant, and therefore it would not pay him to go out at a very small salary; in fact, I do not think nurses are paid sufficient. I have had a nurse at my house at two guineas a week, and he informed me that the four-guinea fee is very seldom charged, and only in cases of an infectious kind; such occasions surely justify the demand for the extra payment. There are numbers of people (not qualified) who are afraid to even look at a room where a patient suffering from a dangerous disease is lying, much more to venture in to attend to the poor sufferer. I consider that nurses are very great friends to sick people, and as such deserving of much more than they get, both male and female, for their work is far from pleasant. How often doctors remark that nothing but thoroughly good nursing will bring a patient round. Well, in those cases it must be the nurse that saves the patient's life, as the doctor does nothing of the nursing, and yet he demands and gets the larger fee.

"Three Years' Man" writes: Your correspondent, "Thirteen Years a Nurse in the Medical Staff Corps," will have it that want of sufficient nursing knowledge is the main reason why doctors will not employ male nurses. I think another reason is that nurses with their nonsense and want of consideration, turn many medical men against them. The fact of the matter is too many of these nurses get beyond their proper place with self-conceit and pretension, and this is only too well-known, and many doctors are deterred from having male nurses on this ground alone. And it is also a fact that doctors prefer women nurses. But this is surely altering, and I do feel that the time is very near when this preference will disappear altogether, and when the whole medical profession will agree that it is only right that men should nurse men. "Thirteen Years a Nurse in the Medical Staff Corps" would fain have us believe him to be an "out and out" nurse. I, for one, hardly think him so, for to my mind the man who is for monopoly is undoubtedly selfish, and so, to say the least of it, a faulty nurse. He would, if he could, rid the Hamilton Association of all the three years' men because they are not experienced enough in nursing. This charge the Secretary (Dr. Cox) can very easily disprove, for these are the nurses who have made the Association, and stuck to it in its darkest times. In conclusion, I would just like to say that it would be difficult, if not nearly impossible, to find a man with three years' service in the Army Medical Staff Corps who is not a practical nurse in every sense of the word. I hope you will give this note a place in your most valuable paper.

### UNIFORMS.

"An Admirer of Outdoor Uniforms" writes: The other day, in The Hospital, you made some remarks on uniform. As a nurse I should like to say that I consider uniform most becoming and useful to nurses, and every trained nurse should wear it. Uniform enables a nurse to go anywhere, and always demands a certain amount of respect; but many of the so-called uniforms we often see now certainly make one wonder where the nurses who wear them were trained, and make one think their garments an invention of their own, with different kinds of kiltings and trimmings added. I suppose all women like to appear to their best advantage, and nurses are only women after all; but let them appear fashionably attired in private, and not try to make outdoor uniform look what it was never intended to be, the "latest fashion" in uniforms. I have worn uniform for eight years, but it is still neat and perfectly plain in style.

### Notes on Novelties.

### EAU DE COLOGNE.

We have received from Messrs. Hockin, Wilson, and Co., 186a, Tottenham Court Road, a bottle of eau de Cologne, for which make they are the sole agents in London. It is very strong in scent, and deliciously refreshing, equalling any we have ever come across, and its moderate price, 10d. for a 2 oz. bottle, and 1s. 8d. for a 4 oz. bottle, will commend it to many people. The Johann Maria Farinas seem to have been a numerous family, if we may judge from the different members of it who make eau de Cologne; they all seem to share a predilection, too, for living near by a "Platz." The maker of this particular brand is Mr. Johann Maria Farina, Gegenuberdem Freisenplatz, Cologne.

### THE ROYAL WARD SHOE.

Yet another firm has taken to making shoes for nurses with the noiseless india-rubber heel. These shoes have rounded toes and a strap across the instep, square heels, with a solid block of rubber under, and they are lined inside with leather. They are only 4s. 11d. a pair, which is a very moderate price if they wear "twelve months," as the advertisement says they will. They may be bought at 296, High Holborn, 96, High Street, Whitechapel, and at other branches of R. and J. Dick's.

### FROM A FELLOW SUFFERER.

The accompanying lines were found in the blotting-book of one who for many years led a life of constant pain, hopeless, it seemed to others, of relief; but she resigned her will into God's hands, and from love and faith in the Saviour gained the peace and rest so ardently longed for. "I want to give a few words of comfort from a suffering soul to other suffering souls, and to assure them that though there is sadness during our earthly pilgrimage, yet joy will come in the morning. On that resurrection morning when our souls will rise with new and glorified bodies to enter into the joy of our Lord." But some will say, how can we hope to come to this happiness, how pass through this time of darkness when Christ's face seems hidden from us, when our painful bodies give us no peace, and our minds are disturbed by the thought that nothing but death can release us from our anguish? Ah! dear friends, believe one who has passed through much tribulation; there is peace and light and rest on this side of the grave, and that it can be reached by treading in the steps which our Lord trod while wearing our mortal flesh. God may have appointed that our pathway here shall be long and sad, that our feet shall be torn with travelling in a sharp and rugged path, but, however dreary our lot, never despair. The burden borne by Him who left His glory for our sakes was far heavier, His way more desolate! Believe me, that, for the sake of our likeness to Him, His loving ear is ever open to our cry, and his heart always ready to answer those who plead the merit of his woes.

Some poor hearts may find the pain of their bodies aggravated by the fear of an offended God; they lie in the gloom of an existence from which he seems shut out, though they earnestly long to return to Him from Whom they have strayed. Know then, dear ones, that He who hung on Calvary's bitter Cross was in deeper darkness of soul that awful day than any other child of man before or since, that He, too, cried in the bitterness of His soul, "My God, my God, why hast Thou forsaken me!" His loving Father was close to Him, though veiled from sight by the sin of the whole world; and He is not far from us either, though we cannot see or feel Him at the moment. Keep on in hope and patient supplication to Him, live in the faith that Christ is in heaven now, that He knows all our anguish of body and of soul, and is always pleading His own death and passion for our relief. Do not dread the morrow, lest it should bring other and stranger trials for which we are unprepared; our heavenly Father will, in His own good time, bid our sufferings cease, and the Lamb of God which taketh away the sins of the world will grant us His peace. It is in the thought of heaven alone that we can find joy and happiness, for

Life is only bright when it proceedeth
Towards a truer, deeper life above;
Human love is sweetest when it leadeth
To a more divine and perfect love.

### An Application for a Vacancy.

She always said she should be a nurse from the time she was a wee thing, and coddled up the lambkins that had lost their mothers, besides looking after all the dogs, cats, and other animals that came to grief on the farm. But as she grew up there were so many things to do at home, and the boys did not want her to go away, and so, what with one thing and another, she put off going to train until she had more leisure to think about it.

And now she really thought she might carry out her long-deferred plans. One of the boys had married and gone, and Millie was getting so useful and such a comfort to father that nobody would miss the eldest daughter from the home. So the small woman made up her mind, and came down to breakfast one morning fully determined to write to one of the big London hospitals that very day.

"Now, Ruth," said her brother Tom, as she handed him his tea without any sugar, having previously put three large lumps into the teapot, "I know you've got hospital fever, and the entire family will suffer. The symptoms are particularly strong this time. You've flattened down the crinkles in your hair, and have assumed that expression of gentle gravity which we know by experience betokens a mind above buttons and tea-cups. But it is of no good," he added, regretfully, as he crunched up a cinder which had got among the fried bacon, "they won't have you, they don't care about girls with snub noses and rough heads at those hospitals. If you were tall and stately, with a die-away sort of voice and sweet ways, there would be a chance for you, but a girl who whistles 'Ta-ra-ra-boom-de-ay,' and calls her brothers names when they try to help her learn natural history and put dear little frogs in her slippers—well! you needn't excite yourself about the prospect."

Then he filled his mouth with as much bread, butter, and lettuce as it would hold, and departed to look after the sheep, while Ruth went about her household tasks without paying much heed to her brother's words.

There seemed so many things to do that morning. Father had asked for a kidney pudding, and Millie's inexperienced hands could not be trusted to make it exactly to his taste. Then that good-for-nothing young boy Dick, the scamp of the family, went and tumbled off a ladder and cut his forehead, and in dressing the wound and the consequent bustle that ensued a good hour passed away. But at last everything seemed settled, the pudding was swelling in the great pot that swung over the broad hearth, the breakfast things were washed up and put away, and Dick, that wretched Dick, was sleeping peacefully on the horse-hair sofa in the parlour, his pains and his sins forgotten.

Ruth had long made up her mind to get her training in nursing at St. Sophia's, and it was to the Matron of that admirable institution that she directed her neat little note, the outcome of much thinking and of much spoiling of writing paper. It was dreadfully difficult to say exactly the right thing in just the right way. The first letter written was too long, taking up no less than three sheets of paper, and giving the little woman's history from babyhood, including an account of the way Dick struggled through the measles and Aunt Jane's recipe for marigold tea. The second was a short note asking simply for information as to a vacancy for a probationer, and seemed on perusal to be too brusque and meagre. The third and fourth, though fairly satisfactory otherwise, contained several blotches and a mistake in spelling. But at last a creditable epistle was achieved, and Ruth, with a sigh of relief, went to find a messenger to send to the post. The boy who usually went on errands was nowhere to be found, but Dick crawled out of the parlour while Ruth was standing on the doorstep looking across the yard. His head was better, he said, and he would take the letter to post; but he must get a drink of water first; so his sister delivered the precious note into his hands, giving him strict injunctions not to drop it, and departed to her neglected poultry-yard, where the hens were all clucking in wonderment at her non-appearance.

Left alone, Dick glanced carelessly at the direction on the envelope he held in his hand. Then he suddenly became interested. St. Sophia's Hospital? that was where lame Jim Smith went last winter, and the doctors had taken all the bones out of his leg, and cleaned them and put them back again, Jim said. What did Ruth want to write to the hospital for? He turned the letter over; it was badly fastened, and, without intending anything wrong, he slipped his thumb under and raised the flap. Then he thought he might as well see what Ruth had written about.

So she wanted to go away from them all, did she? What a shame! Who would make the cakes and tarts and look after a fellow when he got knocked about, and play the harmonium in church, and make jam when the fruit was ripe? Dick, who was very fond of his eldest sister, felt inclined to sit down and howl.

Then suddenly Tom's speech at breakfast flashed into his mind. Perhaps they wouldn't have her; but she had written nothing about her looks and had only mentioned her height and age. "I think it mean of her not to tell them," said Dick reflectively.

The more he thought about it the more sure he was that Ruth ought not to enter St. Sophia's under false pretences.

Finally he tore his sister's neat note into small pieces and put them on the kitchen fire. Then having found, with some difficulty, pen, ink and paper, also a large apple by way of refreshment, he shut himself up in his own room and locked the door. An hour later he might have been seen tearing over the meadow to the post.

This is the letter the astonished Matron of St. Sophia's read the next morning:—

Dear Maddam,—I want to be a nurse in your horspital. I have a snub nose and ruff hair, and can wistle "Tarra-boom-de-a," but not so well as Dick. Sometimes I call my brother an idyot when he teses me. Will you let me know soon, as my little brother is ankyus about me.—Yours affectionitly,

Ruth Willett.

And the crushing answer came back to poor Ruth—

The Matron of St. Sophia's regrets that Miss Willett's qualifications are not those required in a hospital nurse, and advises her to be content with a situation requiring less education and refinement.

Poor little woman, she was too down-hearted to try another hospital, and it was not till six months after, when Dick got kicked by a restive horse and thought he was going to die, that she heard the truth of the matter. The little wretch was too ill to be scolded then, more especially as he had his arm round her neck, and ended with—"But I'm not sorry I did it, 'cause you wouldn't have been here now to look after me; and you may have my clasp-knife and my new pocket-handkerchiefs when I'm dead."

But Ruth did not get these valuable articles; boys like Dick are not killed easily.

## Figures and Tables

**Figure f1:**